# Meteorological Observations

**Published:** 1859-02

**Authors:** 


### METEOROLOGICAL OBSERVATIONS.

We are pleased to be able to resume the publication of our Meteorological Table, with the prospect of its continuance, as the Author, Dr. J. G. Westmoreland, will be engaged in making Observations for the Smithsonian Institute, and will supply us regularly with a transcript of his Monthly Report.

